# Functional Analyses of c.2268dup in Thyroid Peroxidase Gene Associated with Goitrous Congenital Hypothyroidism

**DOI:** 10.1155/2014/370538

**Published:** 2014-03-17

**Authors:** Ching Chin Lee, Fatimah Harun, Muhammad Yazid Jalaludin, Chor Yin Lim, Khoon Leong Ng, Sarni Mat Junit

**Affiliations:** ^1^Department of Molecular Medicine, Faculty of Medicine, University of Malaya, 50603 Lembah Pantai, Kuala Lumpur, Malaysia; ^2^Department of Paediatrics, Faculty of Medicine, University of Malaya, 50603 Kuala Lumpur, Malaysia; ^3^Department of Surgery, Faculty of Medicine, University of Malaya, 50603 Kuala Lumpur, Malaysia

## Abstract

The c.2268dup mutation in thyroid peroxidase (*TPO*) gene was reported to be a founder mutation in Taiwanese patients with dyshormonogenetic congenital hypothyroidism (CH). The functional impact of the mutation is not well documented. In this study, homozygous c.2268dup mutation was detected in two Malaysian-Chinese sisters with goitrous CH. Normal and alternatively spliced TPO mRNA transcripts were present in thyroid tissues of the two sisters. The abnormal transcript contained 34 nucleotides originating from intron 12. The c.2268dup is predicted to generate a premature termination codon (PTC) at position 757 (p.Glu757X). Instead of restoring the normal reading frame, the alternatively spliced transcript has led to another stop codon at position 740 (p.Asp739ValfsX740). The two PTCs are located at 116 and 201 nucleotides upstream of the exons 13/14 junction fulfilling the requirement for a nonsense-mediated mRNA decay (NMD). Quantitative RT-PCR revealed an abundance of unidentified transcripts believed to be associated with the NMD. TPO enzyme activity was not detected in both patients, even though a faint TPO band of about 80 kD was present. In conclusion, the c.2268dup mutation leads to the formation of normal and alternatively spliced TPO mRNA transcripts with a consequential loss of TPO enzymatic activity in Malaysian-Chinese patients with goitrous CH.

## 1. Introduction 

Abnormalities in thyroid hormone synthesis or thyroid dyshormonogenesis accounts for 10 to 20% cases of congenital hypothyroidism (CH), a common endocrine disorder with an incidence of 1 in 2370 to 6000 live births worldwide [1–3]. The remaining 80–85% cases of CH are due to thyroid dysgenesis [4]. Thyroid dyshormonogenesis is linked to defects in genes encoding proteins that are involved in thyroid hormone synthesis, secretion, or recycling [5, 6]. The candidate genes include the thyroid peroxidase (TPO) [7, 8], dual oxidase 2 (DUOX2) [9], solute carrier family 5 or sodium iodide symporter (SLC5A/NIS) [10], and thyroglobulin (Tg) [11]. In some cases, CH patients with dyshormonogenesis develop diffuse or nodular goiter at birth [8, 12, 13] or later in their lives.

TPO gene abnormality is the most common cause of congenital dyshormonogenetic hypothyroidism [14]. To date, more than 60 mutations that affect the TPO activity to varying extents have been described [15]. Among the mutations, the c.2268dup (c.2268 insT) in exon 13 of the TPO gene is a common cause of thyroid dyshormonogenesis among patients in Taiwan and China [16–18]. Ancestors of the studied subjects were believed to have originated from the southern China [16].

Human TPO is a 110 kDA membrane-bound, glycosylated, heme-containing protein that catalyzes the iodination of thyroglobulin and the coupling of iodotyrosyl residues to generate functionally active thyroid hormones, T3 and T4. The single gene encoding TPO is located on chromosome 2p25 which spans at least 150 kb and contains 17 exons [19]. The full length of the TPO mRNA transcript (TPO1) is 3152 bp (GenBank accession number NM_000547.5) in size and codes for a protein consisting of 933 amino acids [20–23].

Apart from TPO1, seven different TPO transcripts which are generated through alternative splicing had also been discovered. Differential splicing generates shorter transcripts: TPO2, TPO3, TPO4, and TPO5 lacking exons 10, 16, 14, and 8, respectively [24–26]. In addition, three other multispliced transcripts such as TPO2/3 (lacking exons 10 and 16), TPO2/4 (lacking exon 10 and 14), and TPO6 that lacks exons 10, 12–14, and 16 had also been described [26]. Among these eight types of TPO transcripts, only TPO1, TPO3, and TPO4 are expected to produce enzymatically active TPO protein and can therefore be expected to play important roles in thyroid hormone synthesis [26, 27]. TPO2, which is lacking in exon 10, leads to a production of TPO protein which does not have any enzymatic activity and is rapidly degraded after synthesis [24]. TPO5 lacks exon 8 which has been suggested to code for a site that participates in catalytic mechanism. The protein translated from TPO5 is also unable to acquire a proper three dimensional configuration and reach the cell surface. In contrast, TPO4 which is lacking in exon 14 is able to fold correctly and reach the cell surface. However, this isoform has a shorter half-life as compared to TPO1 [26].

The c.2268dup mutation was predicted to cause a frame shift that leads to a stop signal after the insertion point [16]. The sequence is then hypothesized to translate into a truncated protein of 756 amino acids that lack the entire transmembrane and intracellular domain which are encoded by exon 15 to exon 17, causing impairment to the expression at the apical membrane [7, 28]. The actual effects of the mutation on the TPO mRNA transcript and the subsequent protein function remain unclear. In this study, we characterized the c.2268dup mutation in two Malaysian-Chinese sisters with goitrous congenital hypothyroidism. The possible effects of the mutation to the TPO mRNA splicing activity, protein function, and expression were also investigated.

## 2. Subjects and Methods

### 2.1. Subjects and Family Members

Two sisters of Malaysian-Chinese origin were diagnosed with CH during infancy. The proband, III-2, was detected to have CH by neonatal screening and the diagnosis was confirmed at 10 days after birth with serum TSH of 120.0 *μ*IU/mL (Normal range: 0.3–5.0 *μ*IU/mL) and free T_4_ of 5.0 pmol/L (Normal range: 20–68 pmol/L). Her elder sister, III-1, was also detected to have CH (but not by neonatal TSH screening as it was not available), at 3 weeks of life with serum TSH of 96.5 *μ*IU/mL and free T_4_ of 2.0 pmol/L when she presented with prolonged jaundice. Both sisters received thyroid replacement with L-thyroxine. Technetium-99m thyroid scintigraphy was performed when they were 3 years old when L-thyroxine was temporarily stopped for 6 weeks and the thyroid function test was repeated. The thyroid scan revealed the presence of thyroid glands despite having a rise in TSH and low free T_4_. In their late teenage years, they developed goiter. Thyroid ultrasonography revealed that both of them had multinodular goiter (MNG). Both sisters had elevated levels of thyroglobulin as the goiters progressed. III-2's thyroglobulin (hTG) level measured when she was 20 years old was 193 ng/mL (reference range 0–55 ng/mL), whilst her elder sister's was much higher at 2288 ng/mL. The proband and her sister (III-1) subsequently underwent total thyroidectomy at the age of 20 years and 24.5 years, respectively. Histology of their thyroid glands revealed the presence of multiple macro- and microfollicular adenomata arising in a background of dyshormonogenetic goiter. The elder sister had a small ventricular septal defect, while the proband had no other congenital anomalies and both of them had normal growth and development. Their parents (II-1 and II-2) who are biologically unrelated were all healthy and euthyroid with no congenital anomalies. However, their maternal grandmother (I-4) was reported to have goiter and had subsequently undergone thyroidectomy with the histology results unknown. Written informed consent was obtained from the parents for their blood and their children's (III-1 and III-2) blood and thyroid tissues for TPO gene analysis. This study was approved by the University of Malaya Medical Centre (UMMC) Ethical Committee (Institutional Review Board) in accordance with the ICH-GCP guideline and the Declaration of Helsinki (Reference number, 654.16).

### 2.2. Screening for c.2268dup Mutation and Other Potential Nucleotide Alterations in the TPO Gene in the Proband, Her Sister, and the Family Members

Genomic DNA was extracted from peripheral venous blood from the proband, the affected sister, and their healthy family members using QIAamp DNA Blood Mini Kit (Qiagen, Germany) according to the manufacturer's protocol. The TPO gene was PCR-amplified with flanking intronic primers covering all the 17 exons [17, 29]. A PCR reaction mixture (50 *μ*L) containing 100–250 ng was prepared in the presence of a final concentration of 1X Taq buffer with KCl, 2.5 mM of MgCl, 200 *μ*M of dNTP, 8% dimethyl sulfoxide (DMSO), 1 unit of Taq DNA polymerase (Fermentas, USA), and 20 pmol of each forward and reverse primers. Thirty-five cycles of amplification were carried out with standard PCR protocol at annealing temperature of 55°C for all coding exons for 30 seconds. The PCR products were purified using QIAquick PCR purification kit (Qiagen, Germany) and then sequenced using ABI Prism Gene Sequencer, Model 3100, Version 3.7 (Research Biolabs, Singapore).

### 2.3. *In Silico* Analysis of the Potential Impact of the c.2268dup on Splicing Activity Using HSF Algorithm

The Human Splicing Finder (http://www.umd.be/HSF/) [30] was used to analyze the existence of alternative splice sites in exon 13 of the TPO gene as a consequence of the c.2268dup mutation. The analysis was done on the whole of exon 13 (171 bp), 50 nucleotides upstream of exon 13 (3′ end of intron 12), and 50 nucleotides downstream of exon 13 (5′ end of intron 13).

### 2.4. Detection of Alternatively Spliced TPO mRNA Transcript Associated with the c.2268dup Mutation

Following thyroidectomy, tissue samples were immediately kept frozen in dry ice and then stored at −80°C. A piece of the pathologically normal tissue was taken near the colloid nodules and the abnormal tissue was taken from the thyroid lesion. Tumors were diagnosed histopathologically and no evidence of malignancy was detected. Each piece of tissue sample of about 30 mg was homogenized and total cellular RNA (tcRNA) was then extracted from the normal and lesionic areas of the thyroid tissues using RNeasy Mini (Qiagen, Germany) according to the manufacturer's instruction. The extracted tcRNA was treated with DNase (Ambion, UK) and then reverse transcribed to complimentary DNA (cDNA) using the High Capacity RNA-to-cDNA Kit (Applied Biosystems, USA). The cDNA was then used as a template to PCR-amplify the coding exons (exons 2–17) using overlapping primers that cover the exon-exon junctions (see Supplementary Table 1 (S1) in the Supplementary Material available online at http://dx.doi.org/10.1155/2014/370538). PCR amplification and the subsequent PCR product purification and sequencing were performed as described earlier except that cDNA was used as a template instead of genomic DNA. Complementary DNA samples prepared from thyroid tissue taken from three individuals without the c.2268dup mutation were used as positive controls.

### 2.5. Quantitation of Normal and Abnormally Spliced TPO mRNA Transcripts in Thyroid Tissues from the Proband and Her Sister

Quantitative real time PCR (qRT-PCR) was used to analyze TPO gene expression in the normal and lesion areas of the thyroid tissues of the proband and her sister. Comparison of the TPO gene expression in thyroid tissues of the proband and her sister was also made. The expression of the TPO gene was normalized to that of a housekeeping gene, the TATA box-binding protein (TBP). In addition, the ratio of TPO mRNA variants in c.2268dup mutation-free individuals was also calculated and compared to the two sisters. Total TPO mRNA transcripts were analyzed using E4F and E5R primer pair covering a region from exons 4 to 5. Primer pairs spanning exons 12 to 13 (E12/13F and E13R) were designed to target normally spliced transcripts, (TPO1, TPO2, TPO3, TPO4, TPO5, TPO2/3, and TPO2/4). Meanwhile, the E12/I12F and E13R primer pair was used to target alternatively spliced-derived transcripts with an insertion of 34 bp between exon 12 and exon 13. TPO6 that does not contain exon 12 and exon 13 was amplified using E11 and E11/15 primers. All qRT-PCR oligonucleotide sequences are listed in Supplementary Table 2 (S2). The qRT-PCR experiments were performed on a StepOnePlus system (Perkin-Elmer/Applied Biosystems). The amplification reaction was carried out using 1X SYBR Green PCR Mastermix (Applied Biosystems, USA), containing 10 ng of cDNA and 200 nM of each primer. The sample was initially denatured at 95°C for 5 min followed by 40 cycles of denaturation at 95°C for 15 sec and subsequent annealing and elongation step at 59°C for 1 min. Melting curves were performed to check the specificity of the amplicons. Each experiment was carried out in triplicate. Comparison of total or target gene mRNA expression was done by calculating the differences in the threshold cycles.

The fold change in expression between tissue samples was calculated by

(1) $$\begin{array}{c} \text{Fold}\;\text{Change}=2^{-\Delta \Delta {\text{C}}_{\text{t}}}, \end{array}$$

where ΔΔC_t_ = (C_t  targeted  TPO  mRNA_ − C_t  TBP_)  lesionic  area − (C_t  targeted  TPO  mRNA_ − C_t  TBP_) normal area.

The fold change in expression between patients was calculated by

(2) $$\begin{array}{c} \text{Fold}\;\text{Change}=2^{-\Delta \Delta {\text{C}}_{\text{t}}}, \end{array}$$

where ΔΔC_t_ = (C_t  targeted  TPO  mRNA_ − C_t  TBP_)  proband − (C_t  targeted  TPO  mRNA_ − C_t  TBP_) proband's sister.

Total 10 ng of cDNA sample from normal and lesionic areas of thyroid tissue (5 ng template from each area) of each patient was used as the template.

Meanwhile, the ratio of TPO mRNA variants to total TPO mRNA in each tissue types was determined by the following calculation.

For normal area of thyroid tissue,

(3) $$\begin{array}{c} \frac{\text{Targeted}\;\text{TPO}\;\text{mRNA}\;{\text{species}}_{\text{normal}\;\text{area}}}{\text{total}\;\text{TPO}\;{\text{mRNA}}_{\text{normal}\;\text{area}}}=2^{-\Delta {\text{C}}_{\text{t}}}. \end{array}$$

For lesionic area of thyroid tissue,

(4) $$\begin{array}{c} \frac{\text{Targeted}\;\text{TPO}\;\text{mRNA}\;{\text{species}}_{\text{lesion}\;\text{area}}}{\text{total}\;\text{TPO}\;{\text{mRNA}}_{\text{lesion}\;\text{area}}}=2^{-\Delta {\text{C}}_{\text{t}}}. \end{array}$$

### 2.6. Microsomal Protein Isolation from Thyroid Tissues

Microsomal protein was isolated from the frozen thyroid tissue specimens. Thirty milligram of tissue was homogenized in ice-cold phosphate buffered saline (PBS) containing Pierce Protease Inhibitors Cocktail (Thermo Scientific, USA) and subsequently centrifuged for 15 min at 3,000 ×g at 4°C to remove nuclei and cytoskeletal components. The supernatant was then ultracentrifuged in the Optima L-100k (Beckman Coulter, USA) at 100,000 ×g for 2 h at 4°C. The pellet was collected and then dissolved in lysis buffer (50 mM Tris-HCl, pH 7.3; 150 mM NaCl; 5 mM EDTA; 10% glycerol (w/v); 1% Triton X-100 (w/v), protease inhibitors) [31]. The mixture was ultrasonicated using Power Sonic 405 (Hwashin Technology, Korea) to improve the yield of the extracted protein.

### 2.7. Immunodetection of TPO Protein

Immunodetection analysis was performed using mouse monoclonal MoAb47 primary antibody (Abcam, UK) and WesternDot 625 Western blot kits (Invitrogen, USA) to detect the presence of TPO protein in the tissue specimens from both patients. Twenty *μ*g of microsomal fraction extract for each sample and 300 ng of a positive control (recombinant fragment of human thyroid peroxidase from Abcam, product code: ab73765, UK) were used in this analysis. The expression of TPO in normal and lesion areas of thyroid tissue from both patients were quantitated by densitometry scanning using Image-J software (http://rsbweb.nih.gov/ij/). The expression of TPO in normal and lesionic areas of the thyroid tissues was normalized to the expression of beta actin. The total expression of TPO protein in the proband was also compared to that of her sister.

### 2.8. Guaiacol Oxidation Assay

Microsomal fraction extracts prepared from patients' thyroid gland tissues were immediately used for enzymatic assay. Microsomes prepared from thyroid tissues from rats were extracted using the same method and were then used as a positive control. Twenty *μ*g of each microsomal fraction were added to 1 mL of reaction mixture containing 40 mM guaiacol (Sigma-Aldrich, USA) and 67 mM sodium phosphate buffer, pH 7.5. The enzymatic reaction was initiated by adding final concentration of 0.25 mM H_2_O_2_. The absorbance was measured at 470 nm (OD_470_) at every second for 3 min at room temperature.

## 3. Results

### 3.1. Mutational Screening of the TPO Gene in the Proband and Her Family Members

**Figure 1:**
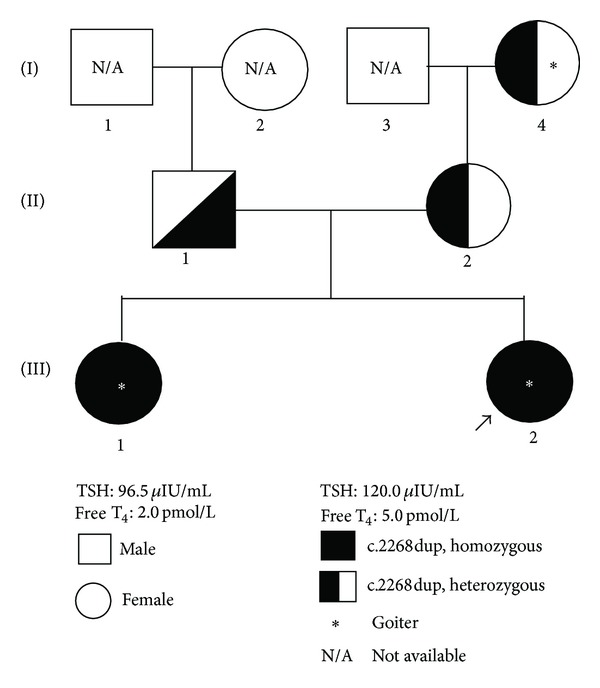
Family pedigree of the index patient. A family pedigree of two Malaysian sisters with congenital hypothyroidism and multinodular goiter. The proband is indicated by the arrow.

PCR amplification followed by DNA sequencing of the TPO gene in the proband, III-2, revealed a homozygous c.2268dup mutation in exon 13. The mutation is predicted to create a premature termination codon at position 757 (p.Glu757x). Further DNA sequencing analysis revealed that her affected sister, III-1, was also homozygous for the same mutation. On the other hand, both parents (II-1 and II-2) and the proband's maternal grandmother (I-4) were found to be heterozygous for the mutation. A family tree of the patient showing the mode of inheritance of the mutation is shown in Figure 1.

### 3.2. HSF Analysis of the Potential Impact of the c.2268dup Mutation on Splicing Activity in Exon 13

HSF analysis predicted that, apart from the natural acceptor and donor splice sites, there are an additional 18 potential acceptor splice sites and 7 potential donor splice sites in exon 13 and the adjacent introns 12 and 13 (50 nucleotides each) of the TPO gene (data not shown). The TPO exon 13 has a strong donor splice site with a consensus value of 94.02 and a weaker acceptor site with a consensus value of 76.12 (Table 1(a)). A potential cryptic acceptor splice site exists at nucleotide position −46 with a stronger score than that of the wild type (83.99 versus 76.12). The mutation, which is located closer to the acceptor site than to the donor site, is predicted by the ESE finder software to disrupt binding sites for three regulatory SR proteins: SRp40, SF2/ASF, and SRp55 at c.2267, c.2268, and c.2269, respectively (Table 1(b)). In addition, ESE motif from HSF algorithm suggested that the c.2268dup mutation creates a new 9 G8 protein binding sites at c.2268 (Table 1(c)).

### 3.3. Detection of Alternatively Spliced TPO mRNA Transcript Associated with the c.2268dup Mutation

**Figure 2:**
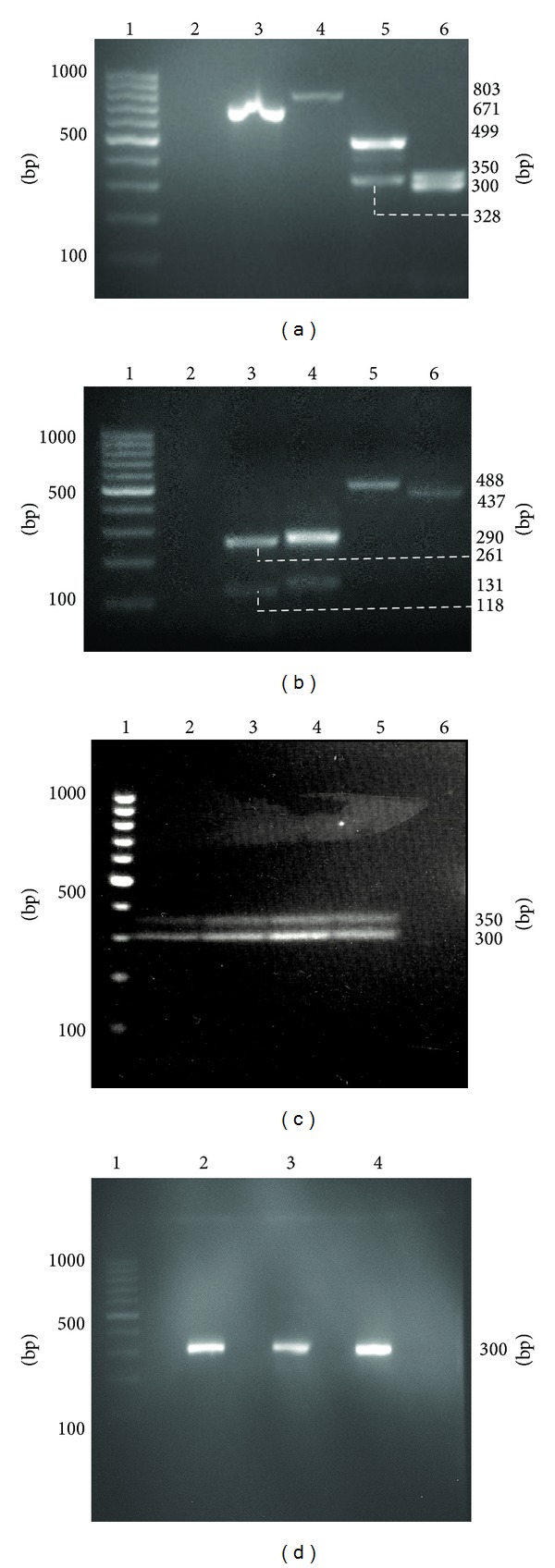
Agarose gel electrophoresis of reverse-transcription PCR (RT-PCR) products. (a) The RT-PCR products of exons 2 to 13 that include the exon-exon junctions. Abnormal spliced products were not detected in exons 2 to 12 based on the expected PCR products present (lanes 3 to 5). However, an unknown product of approximately 350 bp was detected for the exons 12/13 fragment in addition to the expected product of 300 bp. Lane 1: GeneRuler 100 bp DNA Ladder; lane 2: negative control; lane 3: exons 2 to 7, 671 bp; lane 4: exons 7 to 9, 803 bp; lane 5: exons 9 to 12, 499 bp and 328 bp; lane 6: exons 12 to 13, 300 bp and an unknown product of 350 bp. (b)  The RT-PCR products of exons 7 to 9 and exons 13 to 17 that include the exon-exon junctions. Abnormal spliced products were not detected in exons 7 to 9 and exons 13 to 17 based on the expected PCR products present (lanes 3 to 6). Lane 1: GeneRuler 100 bp DNA Ladder; lane 2: negative control; lane 3: exons 13 to 15, 250 bp and 118 bp; lane 4: exons 15 to 17, 261 bp and 131 bp; lane 5: exons 7 to 8, 488 bp; lane 6: exons 8 to 9, 437 bp. (c) The RT-PCR products of exons 12 to 13 that include the exon-exon junction. An unknown product of approximately 350 bp and an expected product of 300 bp were detected in all thyroid tissue specimens from proband and III-1 with c.2268dup mutation. Lane 1: GeneRuler 100 bp DNA Ladder; lane 2: normal area of thyroid tissue of III-1; lane 3: lesion area of thyroid tissue of III-1; lane 4: normal area of thyroid tissue of proband; lane 5: lesion area of thyroid tissue of proband; lane 6: negative control. (d) The RT-PCR products of exons 12 to 13 that include the exon-exon junction. An expected product of 300 bp was detected in all c.2268dup-mutation free individuals.Lane 1: GeneRuler 100 bp DNA Ladder; lane 2: Control I; lane 3: Control II; lane 4: Control III.

RT-PCR analysis of the TPO mRNA transcripts in thyroid tissue of the proband showed two amplicons for exons 9 to 12 (499 bp and 328 bp), exons 12 to 13 (300 bp and 350 bp), exons 13 to 15 (250 bp and 118 bp), and exons 15 to 17 (261 bp and 131 bp) (Figures 2(a) and 2(b)). Sequencing analysis of these RT-PCR fragments confirmed that the extra band detected in exons 9 to 12, exons 13 to 15, and exons 15 to 17 correspond to the TPO2, TPO4, and TPO5 transcripts, respectively (data not shown). Meanwhile, the extra band detected in exons 12 to 13 amplicons contained 34 nucleotides that originate from intron 12. Further analysis showed that this unknown TPO variant was detected in all thyroid issue specimens from both patients (Figure 2(c)) but not in the three c.2268dup mutation-free individuals (Figure 2(d)). The sequence of this novel transcript matched with that of the cryptic acceptor splice (cttttctcgtagTT) in exon 13 (Table 1(a)) that was predicted earlier from the HSF analysis. Apart from the c.2268dup, no other nucleotide alterations were detected in intron 12 and exon 13 of the proband (III-2) and her sister (III-1).

**Figure 3:**
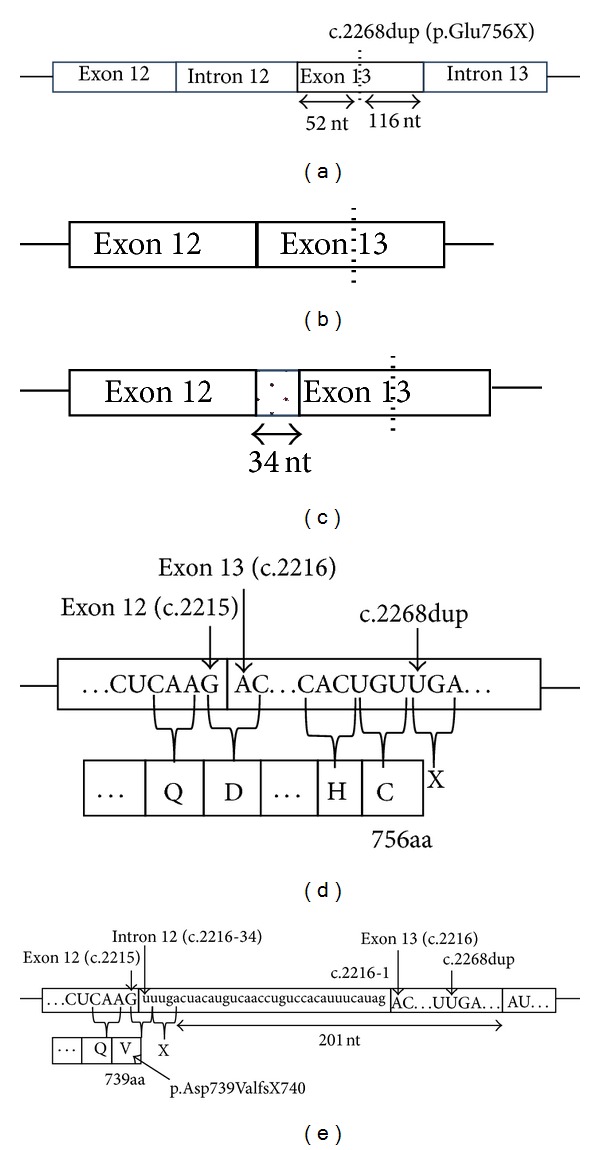
The location and the predicted effects of the c.2268dup mutation. A schematic diagram shows the position of the c.2268dup in genomic DNA (a) and the possible consequences of the mutation on mRNA splicing (b, c) and translation (d, e). Exon sequences are in uppercase; intron sequences are in lower case letters. Deduced protein sequence is shown in one letter code. First in-frame stop codon is represented by a capital letter “X” {Source: http://www.ncbi.nlm.nih.gov/nuccore/NM_000547.5 (NCBI Reference Sequence: NM_000547.5)}.

A schematic diagram shows the position of the c.2268dup in genomic DNA (Figure 3(a)) and the possible consequences of the mutation on mRNA splicing (Figures 3(b) and 3(c)) and translation (Figures 3(d) and 3(e)). As presented by the dotted line in Figure 3(a), the c.2268dup is located at +52 nucleotides (nt) from the exon 12/exon 13 junction and 116 nt upstream of the exon 13/exon 14 junction. The c.2268dup can be predicted to induce a frameshift in the normally spliced product and the appearance of a premature termination codon (PTC) at position 757 p.Glu757X (Figure 3(d)). On the other hand, alternative splicing generating inclusion of 34 nt of intron 12 at the beginning of exon 13 can also be predicted to lead to a frameshift and appearance of PTC at position 740 (p.Asp739ValfsX740).* In silico* analysis using PolyPhen-2 revealed that aspartate at codon 739 was not well conserved (data not shown).

### 3.4. TPO Gene Expression Differences between the Normal and Lesion Areas of Thyroid Tissue of the Proband and Her Sister

**Figure 4:**
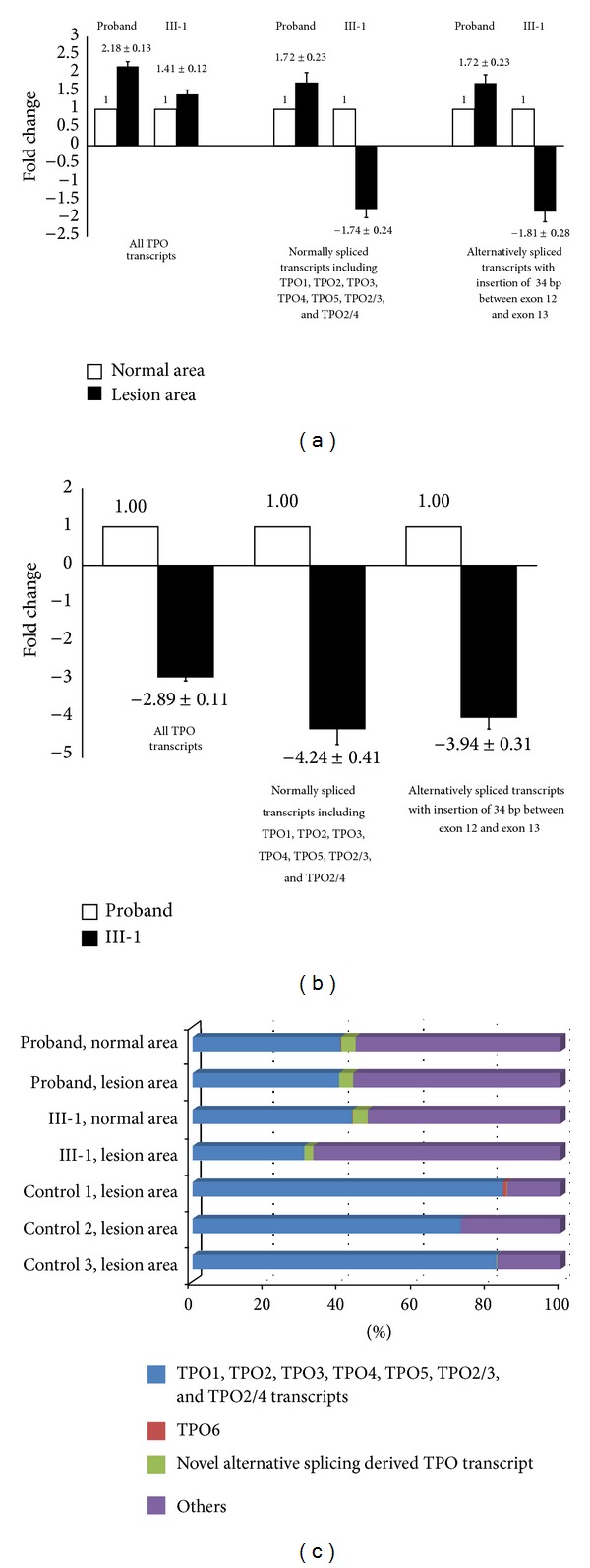
Quantitative real time PCR analysis. Comparison of the TPO gene expression level between the (a) normal and lesion areas of thyroid tissue of proband and III-1, (b) TPO gene expression between proband and III-1, and (c) sum (%) of examined TPO variants in thyroid tissue of proband, III-1, and controls I, II, and III.

The expression of “all TPO mRNA transcripts” in the lesion area of thyroid tissue of the proband was found to be higher than the normal area of the thyroid tissue. The expression of normally spliced TPO mRNA transcripts was concurrent with this result. The level of the alternatively spliced TPO mRNA transcripts was also higher in the lesion compared to the normal area in thyroid tissue of the proband. Compared to the proband, the expression of “all TPO mRNA transcripts” in the lesionic area of thyroid tissue of her affected sister was found to be only slightly higher (<1.5 fold) than that of the normal area. In addition, the total amount of the normally spliced TPO mRNA transcripts and the alternatively spliced transcript was lower than the normal area of the thyroid tissue. The amount of TPO6 in both patients was not able to be determined due to a low yield of PCR product. The relative expression level of TPO mRNA transcripts between the normal and lesionic areas of thyroid tissue of proband and her affected sister is shown in Figure 4(a).

### 3.5. TPO Gene Expression Difference between the Proband and Her Sister

The expression of the TPO gene in III-1 was found to be lower than that of the proband regardless of the type of the TPO transcripts produced. However, the degree of downregulation of the known TPO transcripts including TPO1, TPO2, TPO3, TPO4, TPO5, TPO2/3, and TPO2/4, with (−4.24 ± 0.41) or without normal splicing (−3.94 ± 0.31), is greater than that of the “all TPO mRNA transcripts” (−2.89 ± 0.11). The relative expression level of the TPO mRNA transcripts between the proband and her sister is shown in Figure 4(b).

### 3.6. Quantification of TPO mRNA Variants

The expression ratio of TPO1, TPO2, TPO3, TPO4, TPO5, TPO2/3, and TPO2/4, with or without the 34 nt insertion between exon 12 and exon 13, to the “all TPO mRNA transcripts” in both normal and lesionic areas of thyroid tissue of the proband was similar. The percentage of TPO1, TPO2, TPO3, TPO4, TPO5, TPO2/3, and TPO2/4 in normal and lesionic areas was 40.33 ± 3.41% and 39.94 ± 2.73%, respectively. Meanwhile, the percentage of the alternatively spliced transcript with the insertion of 34 nt was 3.96 ± 0.74% in the normal area and 3.77 ± 0.21% in the area of lesion. Overall, the expression of all known TPO mRNA transcripts, with or without the 34 nt insertion, in normal area was slightly higher than that of the lesionic area. About 55.71% and 56.29% of TPO mRNA transcript in both normal and lesion areas of thyroid tissue of proband was unidentified (unknown TPO transcript). The normally spliced mutant TPO mRNA transcript gave higher level of expression than the alternatively spliced TPO transcript in the ratio of 9.9 : 1 in normal area and 9.5 : 1 in lesionic area.

Compared to the proband, the expression ratio of TPO1, TPO2, TPO3, TPO4, TPO5, TPO2/3, and TPO2/4, with or without insertion of 34 bp between exon 12 and exon 13, to total TPO transcripts in III-1 normal area of thyroid tissue was higher than lesion area of the thyroid tissue. The percentage of normally spliced TPO1, TPO2, TPO3, TPO4, TPO5, TPO2/3, and TPO2/4 in the normal area was 43.45 ± 10.59%, whereas in lesionic area it was only 30.41 ± 5.38%. Meanwhile, the percentage of alternative spliced transcripts in normal and lesionic area was 4.10 ± 0.68% and 2.44 ± 0.28%, respectively. Fifty-two percent and 67% of TPO transcripts in the normal and lesionic areas of thyroid tissue, respectively, belonged to the unknown TPO transcript. The normally spliced mutant TPO transcripts gave higher level of expression than the alternatively spliced TPO transcript in the ratio of 10.6 : 1 in normal area of thyroid tissue and 12.5 : 1 in lesionic area of thyroid tissue.

In contrast, the “all TPO mRNA transcripts” in the c.2268dup mutation-free individuals comprised at least 72.9% of TPO1, TPO2, TPO3, TPO4, TPO5, TPO2/3, and TPO2/4. TPO6 and alternative spliced transcripts with insertion of 34 bp between exon 12 and exon 13 were either detected in small amount (<0.1%) or not detected. The amount of unknown TPO transcripts in the mutation free individuals was estimated in the range of 14.5 to 27%. The sum (%) of all examined TPO variants in c.2268dup mutation in patients and mutation-free individuals is shown in Figure 4(c).

### 3.7. Western Blotting and Guaiacol Oxidation Assay of the Mutant TPO Protein (p.Glu757X)

**Figure 5:**
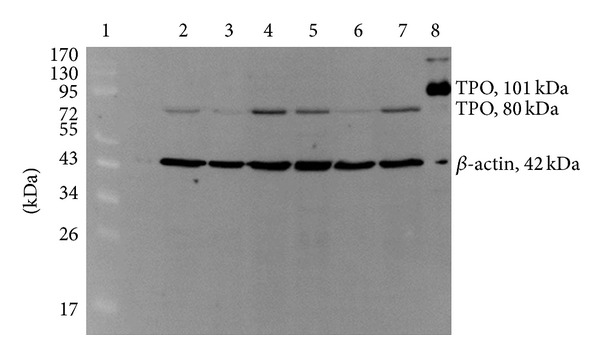
Western blot and Image-J densitometric analyses. Microsomal protein samples were first tested with anti-TPO antibody followed by anti-beta actin antibody (loading control). Equal amount of microsomal protein (20 *μ*g) for each sample (100 ng for the positive control) was used. Lane 1: PageRuler prestained protein ladder; lane 2: normal area of thyroid tissue of proband's sister (III-1); lane 3: lesion area of thyroid tissue of proband's sister (III-1); lane 4: normal area of thyroid tissue of proband; lane 5: lesion area of thyroid tissue of proband; lane 6: normal and lesion areas (10 *μ*g each) of thyroid tissue of proband's sister (III-1); lane 7: normal and lesion areas (10 *μ*g each) of thyroid tissue of proband (III-2); lane 8: recombinant fragment of human thyroid peroxidase (positive control).

Western blot analysis using the MoAb47 primary antibody revealed that the microsomal protein extract from thyroid tissues of both patients contained a faint band of about 80 kDa (Figure 5, lanes 2 to 7). The recombinant fragment of human TPO (positive control) gave a band of 101 kDa (Figure 5, lane 8). Densitometric evaluation of the blots using Image-J algorithm showed that there was a lower expression of TPO protein in the lesion area compared to the normal area of the thyroid tissues. The expression level of the TPO protein found in the lesion area of thyroid tissue of the proband and her sister (III-1) was 1.36-fold and 2.2-fold lower compared to the normal area of their own thyroid tissue. Besides, the amount of TPO protein found in the thyroid tissue of III-1 was significantly lower (6.75 fold) if compared to that of the proband. No peroxidase activity was detected in microsomal protein extracted from thyroid tissues of both patients (data not shown).

## 4. Discussion

TPO abnormality is the most common cause of congenital dyshormonogenetic hypothyroidism [14] where more than 60 mutations in the TPO gene that affect TPO activity to varying extents have been described [15]. Among the mutations, the c.2268dup nonsense mutation in exon 13 of the TPO gene had been reported to be common amongst dyshormonogenetic Taiwanese patients with CH with evidence of a founder effect [16, 17]. The Taiwanese CH patients originated from the same area of Southern Mainland China [17]. In this present study, we identified a homozygous c.2268dup mutation in the TPO gene in two sisters from Malaysian-Chinese family, with CH, with development of goiters later in their lives. Earlier mutational screening excluded thyroid synthesis-related genes, the TSHR and FOXE1, to be the cause of CH and goiter in these two patients [32, 33]. Both parents and the maternal grandmother are all heterozygous for the same mutation.

Studies have suggested that mutations that generate a PTC can disrupt the reading frame and thus result in the production of alternatively spliced mRNAs through a nonsense-associated altered splicing (NAS) mechanism [34]. Other studies have shown that abnormal splicing mechanism can also be triggered as a result of disruption of ESE-binding site sequence by a mutation [35, 36]. In this study, the alternatively spliced TPO transcript with an insertion of 34 bp between exon 12 and exon 13 is postulated to be generated as a result of the interruption of the ESE-binding site sequence in exon 13 of the TPO gene and has abolished the binding motif of three SR proteins, SRp40, SF2/ASF, and SRp55, which are required to strengthen the use of the wild-type (WT) splice site. The alternative splicing mechanism triggered by the c.2268dup has unmasked one of the potential acceptor splice sites by the substitution of the natural acceptor splice site with a sequence motif of cacatttcatagAC located at −12 with an alternative acceptor splice site with a motif of cttttctcgtagTT, located at position −46 generating a sequence of 5′*⋯*AAG/TT*⋯*3′ instead of 5′*⋯*AAG/AC*⋯*3′ at the spliced junction. Sometimes, the alternative splicing mechanism might help to restore the normal reading frame disrupted by a nonsense mutation [12, 37]. However, the alternatively spliced mutant TPO transcript that was generated in this study did not restore the normal reading frame but indeed has led to another stop codon after the 739th amino acid. Despite the activation of a potential splice site in exon 13, the natural acceptor site remains favorable for splicing activity.

In accordance with the mRNA surveillance mechanism, the majority of nonsense-associated transcripts will be degraded via endogenous nonsense-mediated mRNA decay (NMD). However, studies showed that only PTCs that are located at 50–55 nucleotides upstream of an exon-exon junction mediate the mRNA decay. The activation of NMD is believed to limit the expression of abnormal transcript and thus protect the cell from possible harmful effect of the encoded truncated protein [38–42]. In this study, the two PTCs caused by the c.2268dup mutation fulfill the requirement for the mRNA decay, where they are located at 116 and 201 nucleotides away from the exon 13/exon 14 junction. Theoretically, the expression of normally and alternatively spliced mutant transcripts which was reflected by the total amount of PCR amplicon of exons 12/13 should be similar or close to the expression of the total TPO transcripts that was represented by the amplicon of exons 4/5. However, in this study, it was found that the TPO transcripts that contained PTCs due to the c.2268dup mutation were found to be far less abundant compared to the total TPO transcripts. Further analysis showed no evidence of the remaining unidentified TPO transcripts belonged to TPO6 which does not contain exons 10, 12, 13, 14, and 16. To the best of our knowledge, there is no other similar TPO transcript that has been reported so far. Therefore, the abundant amount of unidentified transcripts is believed to be due to (1) partially degraded PTC-containing TPO transcripts or (2) the increase in shorter alternatively spliced TPO isoforms. Under both circumstances, the reduction of functional TPO transcripts could subsequently lead to insufficient TPO protein translation.

In general, the amount of the unidentified TPO transcripts in the lesion area of thyroid tissues of both patients was higher than that of the normal area. The expression of TPO protein in the lesion area of thyroid tissues of both patients was significantly lower than that of the normal area suggesting that the abundant unidentified TPO transcripts were probably degraded and did not get translated, consistent with the hypothesized mRNA surveillance mechanism. The downregulation of TPO expression in lesion area of thyroid tissue and disruption of TPO mRNA maturation has been suggested to be associated with malignant neoplasm [43]. Interestingly, when comparison is made between the proband and her elder sister, III-1, the expression of all known TPO transcripts and the corresponding protein was significantly higher in the former than the latter suggesting that the severity of NMD increases with age. The lower expression of TPO in these two patients may increase the risk of their benign lesion developing into a carcinoma, if the thyroid gland is not removed.

In the early part of this study, a smaller size TPO protein in native form was successfully detected in both patients using MoAb47 antibodies which recognizes an epitope that is located in-between amino acid sequence 713–721 [44]. However, the source of the TPO protein remained unclear since similar size protein can be translated from both normally spliced and alternatively spliced TPO transcripts. The mutant TPO detected in thyroid cells of proband and her sister did not show any enzymatic activity despitethe retention of its important functional sites, suggesting that the absent region is not only important as a hinge for insertion to the membrane but may also play an important role in proper protein folding.

## 5. Conclusion

The c.2268dup mutation leads to the formation of normal and alternatively spliced TPO transcripts with a consequential loss of TPO protein enzymatic activity in the dyshormonogenetic Malaysian-Chinese patients with CH.

## Supplementary Material

Table S1: presents the nucleotide sequence of primers and the expected size of PCR products for mRNA transcript analysis of the TPO gene.
Table S2: presents the nucleotide sequence of primers and the expected size of qRT-PCR products of the TBP (endogenous control) and TPO genes.

## Figures and Tables

**Table 1a:** (a)

Nucleotide position	Splice site type	Motif	New potential splice site	Consensus value (0–100)
−46	Acceptor	cttttctcgtagtt	cttttctcgtagTT	83.99 (CT)
−12	Acceptor	cacatttcatagAC	cacatttcatagAC	76.12 (WT)
169	Donor	AAGgtcagt	AAGgtcagt	94.02 (WT)

CT: cryptic site; WT: wild type.

**Table 1b:** (b)

Nucleotide position	cDNA position	Linked SR protein	Reference motif	Variation*
			(0–100)	(0–100)
51	c.2267	SRp40	TGTGAGG	Site broken
			(78.50)	(−100)
52	c.2268	SF2/ASF	GTGAGGA	Site broken
			(79.27)	(−100)
53	c.2269	SRp55	TGAGGA	Site broken
			(74.82)	(−100)

*Variation expresses the difference between reference and mutant values. Wild-type value is taken as a reference.

**Table 1c:** (c)

Nucleotide position	cDNA position	Linked ESE protein	Reference motif	Variation*
			(0–100)	(0–100)
52	c.2268	9G8	GTTGAG	New site
			(63.49)	(100)

*Variation expresses the difference between reference and mutant values. Wild-type value is taken as a reference.
