# Supplementary material for: Functional Analyses of c.2268dup in Thyroid Peroxidase Gene Associated with Goitrous Congenital Hypothyroidism
Source: Biomed Res Int. 2014 Mar 17;2014:370538. doi: 10.1155/2014/370538 (PMC3976875; doi:10.1155/2014/370538)
Supplement: Supplementary file 1 — Table S1: presents the nucleotide sequence of primers and the expected size of PCR products for mRNA transcript analysis of the TPO gene. Table S2: presents the nucleotide sequence of primers and the expected size of qRT-PCR products of the TBP (endogenous control) and TPO genes. [file 370538.f1.docx]

**Supplementary Table, S1:** Nucleotide sequence of primers and the expected size of PCR products for mRNA transcript analysis of the *TPO* gene. E: Exon; F: Forward primer; R: Reverse primer

| **Amplified region** | **Exons** | **Primer** | **Nucleotide Sequence** | **Fragment size (bp)** | **Exon skipping** |
| --- | --- | --- | --- | --- | --- |
| c.18 to c.688 | 2 - 7 | E2F  E7R | 5’GCTGTCTGTCACGCTGGTTA3’  5’GGAGGTCAGAATAGCGGTCA3’ | 671 | No |
| c.613 to c.1100 | 7 - 8 | E7F  E8R | 5’GTCCGGGAGGTGACAAGAC3’  5’ACGAAGGGCAGGTAGGCG3’ ([29](#_ENREF_29)) | 488 | No |
| c.613 to c.1415 | 7 - 9 | E7F  E9R | 5’GTCCGGGAGGTGACAAGAC3’  5’CCTTCATAGGGACCCACGTA3’ | 803  284 | No  Yes, exon 8 |
| c.979 to c.1415 | 8 - 9 | E8F  E9R | 5’TCCACCGTGTATGGCAGCTC 3’ ([29](#_ENREF_29))  5’CCTTCATAGGGACCCACGTA3’ | 437 | No |
| c.1572 to c.2070 | 9 -12 | E9F  E12R | 5’CAGCCCATGGACATTACTCC3’  5’GGAGTGCTTCTCCAGCTCAC3’ | 499  328 | No  Yes, exon 10 |
| c.2071 to c.2370 | 12 - 13 | E12F  E13R | 5'-CTGTCTCGGGTCATCTGTGA-3’  5'-CTGGAAATCCCATCCTTCCT-3’ | 300 | No |
| c.2351 to c.2600 | 13 - 15 | E13F  E15R | 5’AGGAAGGATGGGATTTCCAG3’  5’GAGGTGAGACCTGCGAAGC3’ | 250  118 | No  Yes, exon 14 |
| c.2551 to c.2793 | 15 - 17 | E15F  E17R | 5’ CCTCGGGTGACTTGGATCT3’  5’ TCTCGGCAGCCTGTGAGTATC3’([26](#_ENREF_26)) | 261  131 | No  Yes, exon 16 |

**Supplementary Table, S2:** Nucleotide sequence of primers and the expected size of qRT-PCR products of the *TBP* (endogenous control) and *TPO* genes. E: Exon; I: Intron; F: Forward primer; R: Reverse primer; *([NM_003194.4](http://www.ncbi.nlm.nih.gov/entrez/viewer.fcgi?db=nucleotide&id=285026518) )

| Genes | Amplified region | Exon | Intron | Primer | Nucleotide Sequence | Fragment size (bp) |
| --- | --- | --- | --- | --- | --- | --- |
| *TBP** | c.620 to c.708 ([43](#_ENREF_43)) | 4 - 5 | - | E4F  E5R | 5’-CACGAACCACGGCACTGATT-3’  5’-TTTTCTTGCTGCCAGTCTGGAC-3’ | 89 |
| *TPO* | c.229 to c.469 | 4 - 5 | - | E4F  E5R | 5’-TCCAAACTTCCTGAGCCAAC-3’  5’-CTCCTGTGATGGGCCTGTAT-3’ | 241 |
| *TPO* | c.2207 to c.2370 | 12 - 13 | - | E12/E13F  E13R | 5’-TTCCTCAAGACGACAAGTGTG-3’  5'-CTGGAAATCCCATCCTTCCT-3’ | 164 |
| *TPO* | c.2203 to c.2214...c.2216-34 to c.2370 | 12 - 13 | 12 | E11/I12F  E13R | 5’-ACCTTTCCTCAAGtttgactac-3’  5'-CTGGAAATCCCATCCTTCCT-3’ | 202 |
| *TPO* | c.1905 to c.2008  ...c.2519 to c.2526 ([43](#_ENREF_43)) | 11,15 | - | E11F  E11/E15R | 5’- GCTGGGAGGCTTAGTTGAAA-3’  5’- CCCGGAGTACCAGTCACCAT-3’ | 112 |
